# CT radiomics with transfer learning features for detecting DECT−positive periarticular monosodium urate crystal deposition: a single−center retrospective study

**DOI:** 10.3389/fendo.2026.1881194

**Published:** 2026-07-01

**Authors:** Weitao Huang, Xingjian Xu, Yongjun Ye, Yuguo Wei, Wenqiang Zheng, Xiaowei Han, Guozheng Zhang

**Affiliations:** 1 Department of Radiology, Quzhou People’s Hospital; The Quzhou Affiliated Hospital, Wenzhou Medical University, Quzhou, China; 2 Zhejiang Chinese Medical University, Hangzhou, China; 3 The Fifth Affiliated Hospital of Wenzhou Medical University, Lishui, China; 4 Pharmaceutical Diagnosis, GE Healthcare, Hangzhou, China

**Keywords:** artificial intelligence, computed tomography, deep learning, gout, monosodium urate, radiomics, transfer learning, urate crystal deposition

## Abstract

**Objective:**

To develop and validate single−energy CT (135 kVp)−based radiomics and deep learning models for the non−invasive detection of periarticular monosodium urate (MSU) crystal deposition.

**Methods:**

This retrospective study included 605 patients with suspected periarticular MSU deposition, randomly split into a training cohort (n=425) and a validation cohort (n=180). Clinical variables and CT values were collected. Hand−crafted radiomics features were extracted from lesion ROIs and selected using t−test, Pearson correlation, LASSO, and mRMR. Deep features were derived from the maximum cross−sectional ROI using a ResNet50 transfer learning framework, and fused features underwent the same selection. A multilayer perceptron was used to construct the radiomics, deep learning radiomics (DLR), and combined (clinical + DLR) models, as well as a clinical−only model.

**Results:**

Serum uric acid (OR 1.003), age (OR 1.017), bone erosion (OR 3.476), and CT value (OR 0.993) were independently associated with MSU deposition (all *P* < 0.05). AUCs for the validation cohort were 0.820 (clinical), 0.912 (radiomics), 0.940 (DLR), and 0.942 (combined). The combined model achieved accuracy 0.889, sensitivity 0.905, and specificity 0.837. The DLR and combined models significantly outperformed the clinical model (DeLong *P* < 0.05), with no significant difference between them.

**Conclusions:**

The single−energy CT−based radiomics and deep learning radiomics models showed comparable performance for identifying periarticular MSU deposition (no statistically significant difference). The combined clinical−imaging model achieved numerically higher performance but did not significantly outperform the deep learning radiomics model.

## 1 Introduction

Gout is a common metabolic crystal arthropathy caused by the deposition of monosodium urate (MSU) crystals in joints and periarticular tissues. Gout is closely associated with metabolic comorbidities such as hyperuricemia, chronic kidney disease, hypertension, diabetes mellitus, and cardiovascular disease (1, 2). The detection of MSU crystals in synovial fluid or tophus aspirate is the reference standard, but arthrocentesis is invasive, operator−dependent, and not well accepted (3, 4). Serum uric acid alone is insufficient for diagnosis (5). Consequently, imaging plays an increasingly important role, especially for early or atypical presentations.

Currently available imaging modalities each have strengths and limitations. Radiography is widely accessible but insensitive to early urate deposition. Ultrasound can detect the double contour sign and tophi but is operator−dependent (6, 7). Magnetic resonance imaging has high soft−tissue resolution but lacks specificity (8). Dual−energy CT (DECT) can directly color−code urate deposition and has become an important non−invasive tool (9, 10), with meta−analyses reporting good diagnostic performance (11, 12). However, DECT equipment is not universally available, and its sensitivity is limited for early or non−tophaceous deposits (13, 14). Therefore, developing quantitative diagnostic tools based on single−energy CT (acquired at 135 kVp) —which is more widely accessible—has practical relevance.

Radiomics extracts high−dimensional quantitative features from medical images (15, 16), while deep learning, especially transfer learning with pretrained networks (e.g., ResNet50), can capture complex image representations (17, 18). Hand−crafted radiomics features and deep learning features may be complementary, but their relative performance for detecting MSU deposition on single−energy CT (135 kVp) has not been systematically compared.

The novelty of this study is threefold. First, to our knowledge, this is the first study to combine hand−crafted radiomics features with ResNet50−based transfer learning features extracted from standard single−energy CT images for detecting periarticular MSU deposition. Second, we systematically compared four models (clinical, conventional radiomics, deep learning radiomics, and combined clinical−imaging) to determine whether deep transfer learning provides additional value over hand−crafted features. Third, we explicitly restricted the reference standard to DECT−positive deposits and positioned the model as a screening aid rather than a diagnostic replacement, addressing a practical gap in settings where DECT or joint aspiration is unavailable.

Against this background, this study aimed to develop and validate standard single−energyCT−based radiomics and deep learning models for detecting DECT−positive periarticular MSU crystal deposition. We hypothesized that models built on quantitative CT image features would outperform a purely clinical model, and that the combined model would achieve the best diagnostic performance.

## 2 Materials and methods

### 2.1 Patients

Approval for this study was obtained from the Medical Ethics Committee of Quzhou People’s Hospital (Approval NO: 2025-164). A total of 605 patients who underwent CT evaluation for suspected periarticular MSU crystal deposition were included. The outcome label was determined according to the reference standard in the source data. In this study, the reference standard was defined as urate deposition identified by dual-energy CT (DECT), which served as an imaging reference rather than the gold standard for gout diagnosis. It should be explicitly noted that the currently accepted gold standard remains the detection of monosodium urate (MSU) crystals in synovial fluid or tophus aspirate using polarized light microscopy. Given the limited sensitivity of DECT for non−tophaceous lesions, the results of this study should be interpreted with this limitation in mind. The unit of analysis was one lesion per patient. For patients with multiple affected joints or multiple lesions, only the most representative joint and the largest lesion were selected for analysis. Therefore, all included cases were independent at the patient level. All patients were randomly divided into a training cohort and a validation cohort at a 7:3 ratio, with 425 patients in the training cohort and 180 in the validation cohort.

**Figure 1:**
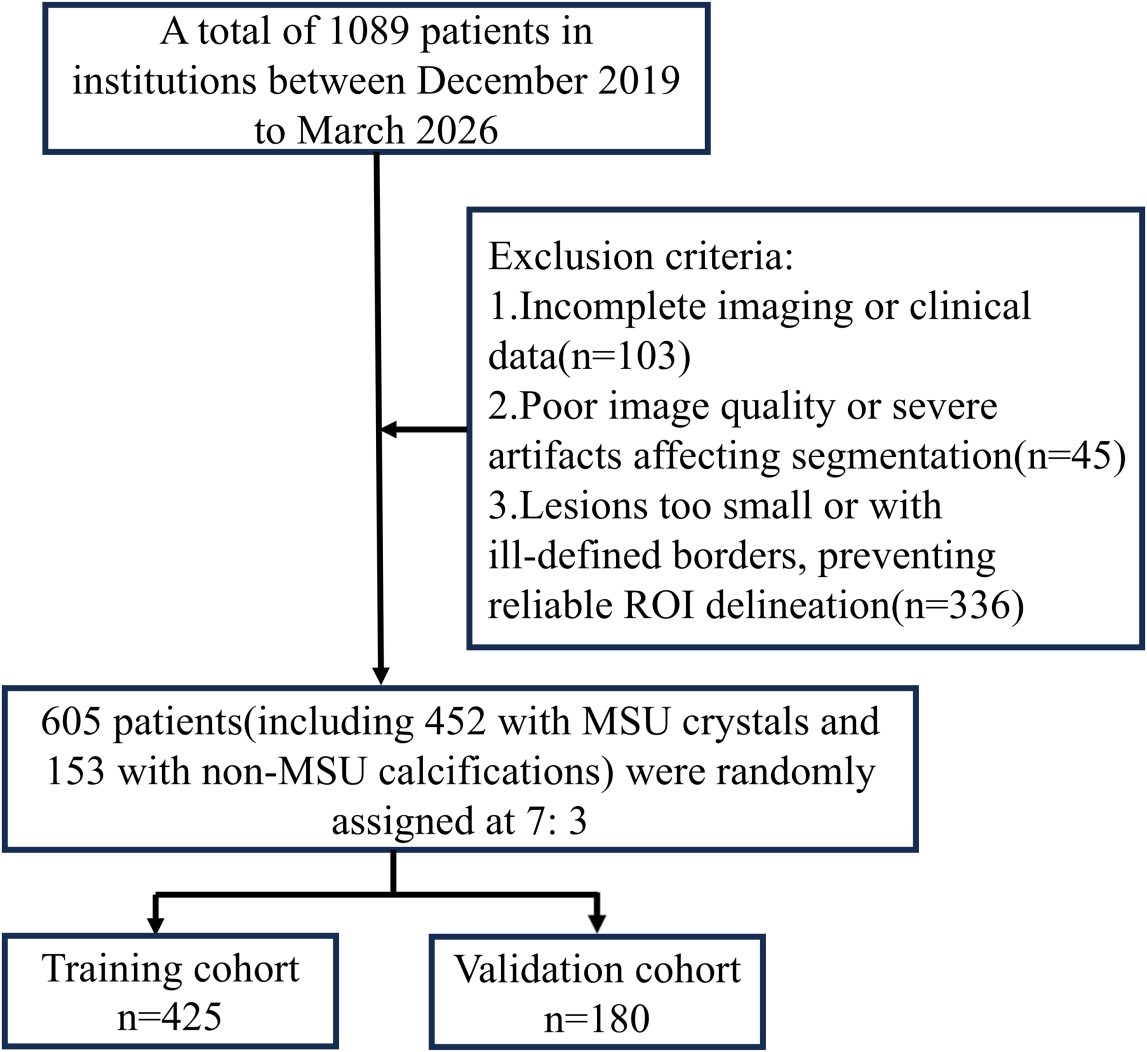
Flow chart of study inclusion.

Inclusion criteria were: availability of analyzable CT images, complete clinical data, and a definitive reference label for periarticular MSU crystal deposition. Exclusion criteria were: incomplete imaging or clinical information, poor image quality or severe artifacts compromising segmentation, and lesions that were too small or had ill-defined borders precluding reliable region-of-interest delineation. The case selection process is shown in **Figure 1**. Non−MSU status was confirmed by DECT post−processing analysis; the specific etiologies of non−MSU calcifications were heterogeneous and not further sub−classified.

### 2.2 Clinical data collection and CT image assessment

Baseline clinical and CT variables were extracted from the source data, including age, sex, CT value, serum uric acid, bone erosion, hypertension, diabetes mellitus, history of alcohol consumption, and history of nephrolithiasis. CT images were reviewed using standardized window width and window level settings before entering the post-processing pipeline. Bone erosion was recorded as a binary sign based on standard single−energyCT findings.

### 2.3 Image acquisition and ROI segmentation

Image acquisition was performed using a Toshiba Aquilion ONE TSX−301C 320−slice dual−energy CT scanner. The scanning parameters were: dual-energy mode, tube voltages of 135 kV and 80 kV, tube current modulation using automatic control (Care Dose 4D technology), gantry rotation time of 0.275 s, reconstruction slice thickness of 0.5 mm, and slice interval of 0.5 mm. All CT images were transferred to a workstation for review and analysis, with DICOM-format images sourced from the Picture Archiving and Communication System (PACS). For image pre-processing, a window width of 1600 HU and a window level of 400 HU were set to optimize the contrast between urate deposits and surrounding soft tissues.

Although the scanner operates in dual−energy mode, the images used for radiomics and deep learning feature extraction were the 135 kV single−energy image series, not the post−processed mixed images or other dual−energy parameters. The 135 kV tube voltage is a commonly used high−energy setting in routine CT examinations, and the image characteristics (CT numbers, noise, contrast) obtained at this voltage are comparable to those acquired on non−DECT single−energy CT scanners using similar voltages (120–140 kV). Therefore, the model is in principle applicable to standard single−energy CT devices, but we acknowledge that no external validation on truly independent standard single−energyCT scanners was performed.

Region of interest (ROI) delineation was performed using the open-source software ITK-SNAP (version 3.4). Two radiologists with more than five years of experience in musculoskeletal imaging independently and manually delineated ROIs slice by slice along the lesion margins. During delineation, images were magnified as much as possible, and care was taken to avoid including adjacent calcifications and articular bone structures, ensuring that the ROI primarily represented urate deposits or suspicious lesional areas. In cases of disagreement between the two radiologists, consensus was reached through consultation with a third senior radiologist with 15 years of diagnostic experience. The intraclass correlation coefficient (ICC) was used to assess inter- and intra-observer agreement for ROI segmentation. A random subset of 30 patients was selected for reproducibility analysis. The inter-observer ICC for ROI segmentation was 0.94 (95% CI: 0.91–0.96), and the intra-observer ICC (with a two-week interval) was 0.92 (95% CI: 0.88–0.95), both indicating excellent agreement (ICC > 0.75 was considered indicative of good agreement).

### 2.4 Radiomics and deep learning feature extraction

**Figure 2:**
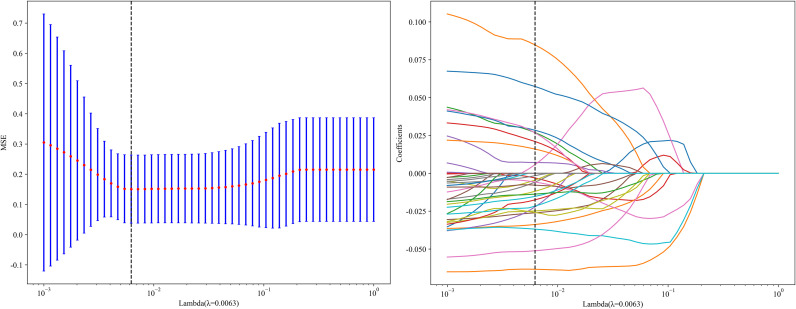
Hand-crafted radiomics feature selection using the least absolute shrinkage and selection operator (LASSO) and the histogram of radiomics feature importance scores based on the selected features. The optimal λ value of 0.0063 was selected.

**Figure 3:**
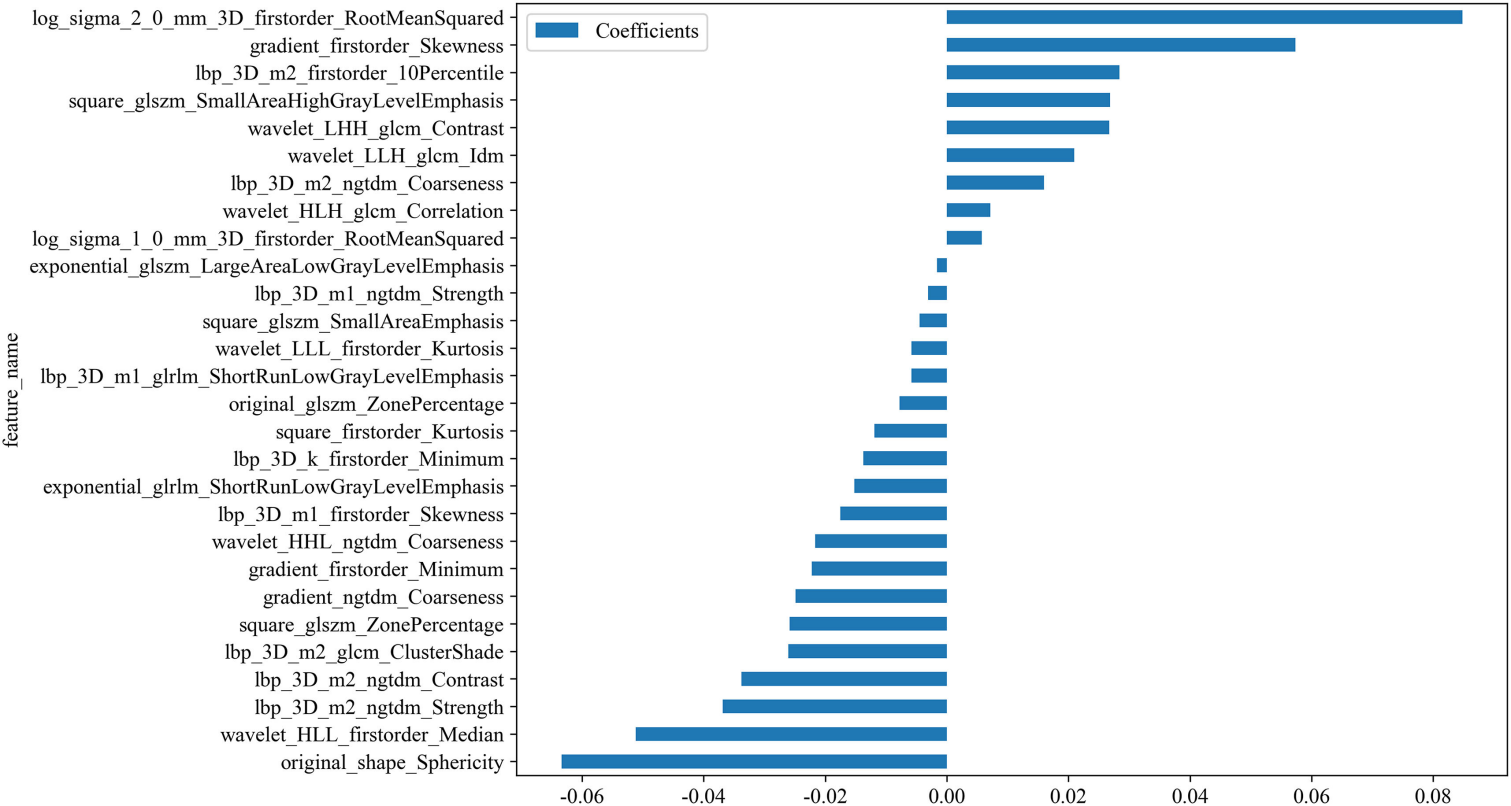
Weight values of the features included in the final radiomics signature. Each bar represents the coefficient of a retained hand-crafted radiomics feature, indicating its relative contribution to the signature.

Hand-crafted CT radiomics analysis was performed based on the lesion ROIs, yielding a total of 1834 hand-crafted radiomics features, primarily including shape features, first-order statistical features, texture features, and features derived from filtered transformed images. Radiomics feature extraction was performed using PyRadiomics version 3.0.1, following the Image Biomarker Standardization Initiative (IBSI) guidelines. Prior to extraction, ROIs were resampled to isotropic voxel size of 1×1×1 mm³ using B−spline interpolation. Gray−level intensities were discretized using a fixed bin width of 25 Hounsfield units (HU). In addition to the original image, seven filter types were applied: wavelet, Laplacian of Gaussian (LoG, sigma values 1.0, 2.0, 3.0), square, square root, logarithm, exponential, and gradient. No additional image normalization was applied because CT numbers are standardized absolute values. To reduce dimensionality and mitigate overfitting, all feature selection was conducted exclusively within the training cohort using a stepwise strategy comprising (1): univariate t-test to screen features associated with the outcome (*p* < 0.05). This step retained 1,342 out of 1,834 hand−crafted features (2). Pearson correlation analysis to remove highly correlated features with an absolute correlation coefficient > 0.9; after this step, 758 features remained (3). LASSO regression with ten−fold cross−validation to select features with non−zero coefficients; the optimal penalty parameter λ (0.0063, as shown in **Figure 2**) was determined by minimum cross−validation error, yielding 64 features (4). mRMR (mutual information maximization) to select the top 20 most relevant and least redundant features (k = 20). The same four−step selection pipeline was applied to the fused features (hand−crafted + deep learning features). The parameter settings (Pearson threshold 0.9, mRMR k = 20, LASSO λ via cross−validation) were chosen based on prior radiomics literature and were fixed before model training to avoid data leakage. We acknowledge that a multi−step selection pipeline carries a risk of over−engineering; however, all selection was locked to the training cohort only, and the final model performance was evaluated on the independent validation cohort without further feature tuning. The final radiomics signature was constructed as a linear combination of the selected features weighted by their coefficients. The weights of the features retained in the final radiomics signature are displayed in **Figure 3**.

Before deep learning feature extraction, the maximum cross-sectional ROI was selected, cropped, and resampled to 64 × 64 pixels. Pixel intensities were normalized to a mean of 0 and a standard deviation of 1. A transfer learning framework based on ResNet50 was employed as the feature extractor. Model training used the stochastic gradient descent optimizer with a batch size of 64, 50 epochs, and an initial learning rate of 0.01. After training was completed, the model parameters were frozen, and the penultimate layer was taken as the deep learning feature layer. Ultimately, 2024 deep learning features were obtained per case. Methodologically, the transfer learning pipeline and quantitative CT modeling strategy in this study primarily drew on previous studies on transfer learning for medical imaging and CT radiomics of uric acid-related diseases (17–19).

**Figure 4:**
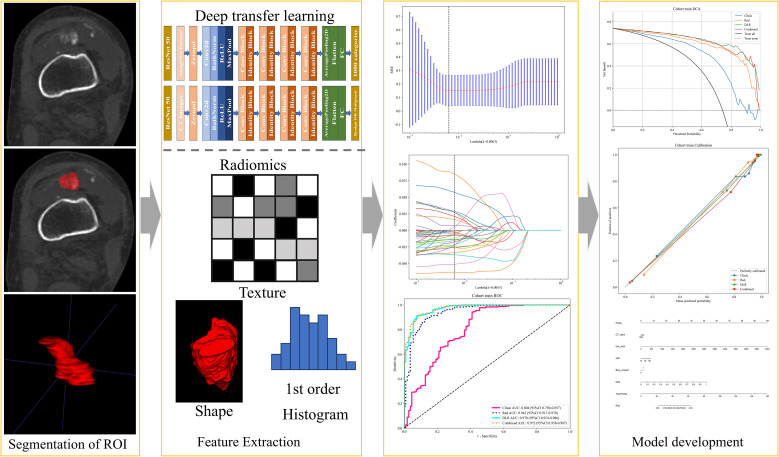
Deep learning radiomics workflow.

The deep learning radiomics pipeline fused the 1834 hand-crafted radiomics features with the 2024 deep learning features to form 3858 fused features, which were then subjected to the same feature selection and dimensionality reduction approach as used for the radiomics model. Key steps in the radiomics analysis process included CT image acquisition, lesion segmentation, feature extraction and selection, and model construction (**Figure 4**).

ImageNet-pretrained ResNet50. Single−channel CT images duplicated to three channels. ROIs resized to 64×64, normalized to mean=0, std=1. No augmentation. Frozen conv layers, fine−tuned FC. Binary cross−entropy loss, class weights. SGD (lr=0.01, momentum=0.9, weight decay=1e-4, batch size=64, 50 epochs), early stopping (patience=10). Fixed random seed. PyTorch. Features from global average pooling (2048 dim) followed by a linear layer to 2024 dim.

### 2.5 Model construction and evaluation

This study first compared logistic regression, extremely randomized trees, and a multilayer perceptron for constructing the radiomics model. Based on their overall performance in the data, the multilayer perceptron was selected for subsequent modeling.

Four models were ultimately built: a clinical model based on clinical and CT variables; a radiomics model based on radiomics features; a deep learning radiomics model based on fused radiomics and deep transfer learning features; and a combined model integrating clinical variables and the deep learning radiomics signature.

Model performance was evaluated using accuracy, AUC, 95% confidence interval, sensitivity, specificity, positive predictive value, negative predictive value, precision, recall, F1 score, and threshold. Pairwise model comparisons were performed with the DeLong test, and incremental discriminatory ability was quantified using net reclassification improvement (NRI) and integrated discrimination improvement (IDI). Calibration curves and decision curve analysis were used to assess the agreement between predicted probabilities and observed outcomes and the potential net clinical benefit, respectively. Gradient-weighted class activation mapping was employed to visualize the image regions on which the deep model focused.

Model training details: For the multilayer perceptron (MLP) classifier used in the radiomics, DLR, and combined models, the network architecture consisted of three hidden layers with 128, 64, and 32 neurons respectively, each followed by ReLU activation and dropout (rate = 0.3). The model was trained using the Adam optimizer (learning rate = 0.001, β1 = 0.9, β2 = 0.999) with a batch size of 32. Early stopping was applied based on the validation loss with a patience of 10 epochs. The maximum number of epochs was set to 200, and the final model was selected at the epoch with the lowest validation loss. For the deep learning feature extractor (ResNet50), transfer learning was performed as described in section 2.4, with stochastic gradient descent (SGD) optimizer (learning rate = 0.01, momentum = 0.9, weight decay = 1e−4), batch size of 64, and 50 epochs. All model training was performed exclusively on the training cohort, and hyperparameters were tuned using five−fold cross−validation within the training cohort. No validation cohort data were used for feature selection or hyperparameter tuning.

### 2.6 Statistical analysis

Continuous variables are presented as mean ± standard deviation, and categorical variables are presented as frequencies and percentages. The balance of baseline characteristics between the training and validation cohorts was compared. Continuous variables were compared using the independent-samples *t*-test or non-parametric tests depending on the data distribution, and categorical variables were compared using the chi-square test or Fisher’s exact test. Univariate and multivariate logistic regression analyses were used to screen clinical and standard single−energyCT factors associated with periarticular MSU crystal deposition. Clinically meaningful variables that were significant in univariate analysis were entered into the multivariate analysis. A two-sided *P* < 0.05 was considered statistically significant.

## 3 Results

### 3.1 Patient baseline characteristics

A total of 605 patients were included, with 425 in the training cohort and 180 in the validation cohort. The overall mean age was 57.61 ± 17.12 years, mean CT value was 343.95 ± 224.33 HU, and mean serum uric acid level was 486.32 ± 257.76 µmol/L. Bone erosion was present in 304 patients (50.25%). Hypertension, diabetes mellitus, history of alcohol consumption, and history of nephrolithiasis were found in 213 (35.21%), 76 (12.56%), 166 (27.44%), and 147 (24.30%) patients, respectively.

Baseline characteristics were well balanced between the training and validation cohorts. No statistically significant differences were found between the two groups in age (57.59 ± 17.11 vs. 57.67 ± 17.21 years, *P* = 0.961), CT value (343.94 ± 224.01 vs. 343.96 ± 225.70 HU, *P* = 0.831), serum uric acid (491.94 ± 293.73 vs. 473.04 ± 139.92 µmol/L, *P* = 0.524), sex distribution (*P* = 0.548), bone erosion (*P* = 0.729), hypertension (*P* = 0.560), diabetes mellitus (*P* = 0.765), history of alcohol consumption (*P* = 0.982), or history of nephrolithiasis (*P* = 0.798) (**Table 1**).

**Table 1:** Baseline characteristics of the study cohorts.

Variable	Overall (n=605)	Training cohort (n=425)	Validation cohort (n=180)	P-value
Age, years	57.61 ± 17.12	57.59 ± 17.11	57.67 ± 17.21	0.961
CT value, HU	343.95 ± 224.33	343.94 ± 224.01	343.96 ± 225.70	0.831
Serum uric acid, μmol/L	486.32 ± 257.76	491.94 ± 293.73	473.04 ± 139.92	0.524
Male sex, n (%)	564 (93.22)	394 (92.71)	170 (94.44)	0.548
Bone erosion, n (%)	304 (50.25)	216 (50.82)	88 (48.89)	0.729
Hypertension, n (%)	213 (35.21)	146 (34.35)	67 (37.22)	0.560
Diabetes mellitus, n (%)	76 (12.56)	55 (12.94)	21 (11.67)	0.765
Alcohol use, n (%)	166 (27.44)	116 (27.29)	50 (27.78)	0.982
Nephrolithiasis, n (%)	147 (24.30)	105 (24.71)	42 (23.33)	0.798

Continuous variables are presented as mean ± standard deviation; categorical variables as n (%). HU, Hounsfield unit.

### 3.2 Clinical factor selection using univariate and multivariate analysis

Univariate analysis showed that CT value, serum uric acid, age, diabetes mellitus, hypertension, sex, history of alcohol consumption, bone erosion, and history of nephrolithiasis were all associated with the outcome. After multivariate adjustment, serum uric acid, age, bone erosion, and CT value remained independent factors associated with periarticular MSU crystal deposition.

Serum uric acid was an independent positive predictor (OR 1.003, 95% CI 1.001–1.004, *P* = 0.002). Age was also independently associated with the outcome (OR 1.017, 95% CI 1.005–1.028, *P* = 0.017). Among standard single−energyCT signs, bone erosion showed the strongest association (OR 3.476, 95% CI 2.046–5.906, *P* < 0.001). CT value was also identified as a multivariate predictor (OR 0.993, 95% CI 0.991--0.994, P < 0.001). However, the OR is extremely close to 1, corresponding to a negligible effect size (approximately 0.7% risk reduction per one-unit increase in HU). Moreover, the direction of the association reversed from the univariate analysis (OR 1.001), likely due to confounding or multicollinearity. Therefore, this finding should be interpreted with caution and its clinical significance is limited. Diabetes mellitus, hypertension, sex, history of alcohol consumption, and history of nephrolithiasis did not reach statistical significance in the multivariate model (**Table 2**).

**Table 2:** Predictors of monosodium urate crystal deposition: univariate and multivariate logistic regression.

Variable	Univariate OR (95% CI)	P-value	Multivariate OR (95% CI)	P-value
CT value	1.001 (1.000–1.001)	0.022	0.993 (0.991–0.994)	<0.001
Serum uric acid	1.002 (1.002–1.003)	<0.001	1.003 (1.001–1.004)	0.002
Age	1.018 (1.015–1.021)	<0.001	1.017 (1.005–1.028)	0.017
Diabetes mellitus	2.235 (1.383–3.611)	0.006	0.996 (0.449–2.208)	0.993
Hypertension	2.561 (1.891–3.466)	<0.001	0.647 (0.361–1.160)	0.220
Male sex	3.020 (2.494–3.658)	<0.001	2.269 (1.099–4.683)	0.063
Alcohol use	3.640 (2.512–5.275)	<0.001	1.652 (0.896–3.047)	0.177
Bone erosion	5.171 (3.815–7.008)	<0.001	3.476 (2.046–5.906)	<0.001
Nephrolithiasis	6.000 (3.792–9.488)	<0.001	2.188 (1.123–4.259)	0.053

OR, odds ratio; CI, confidence interval.

### 3.3 Radiomics signature and classifier comparison

After dimensionality reduction and selection, the final radiomics signature was composed of multiple hand-crafted radiomics features, including shape, first-order statistics, gray-level co-occurrence matrix, gray-level size zone matrix, gray-level run-length matrix, and neighborhood gray-level difference matrix features. This signature integrated intensity, morphological, and textural information that is difficult to fully capture by conventional visual assessment.

Among the three candidate machine learning algorithms, logistic regression achieved AUCs of 0.913 and 0.921 in the training and validation cohorts, respectively; extremely randomized trees achieved 0.885 and 0.898; the multilayer perceptron achieved an AUC of 0.943 in the training cohort and 0.912 in the validation cohort, and delivered the highest accuracy (0.917), sensitivity (0.964), negative predictive value (0.868), recall (0.964), and F1 score (0.946) in the validation cohort. Therefore, the multilayer perceptron was selected for subsequent model construction (**Table 3**).

**Table 3:** Performance of radiomics models using three machine learning classifiers.

Model	Cohort	Accuracy	AUC (95% CI)	Sensitivity	Specificity	PPV	NPV	F1-score	Threshold
LR	Training	0.866	0.913 (0.879–0.948)	0.895	0.782	0.922	0.723	0.908	0.706
LR	Validation	0.878	0.921 (0.872–0.969)	0.883	0.860	0.953	0.698	0.917	0.682
ET	Training	0.880	0.885 (0.844–0.926)	0.914	0.782	0.923	0.761	0.919	0.663
ET	Validation	0.806	0.898 (0.839–0.957)	0.766	0.930	0.972	0.556	0.857	0.754
MLP	Training	0.882	0.943 (0.917–0.970)	0.886	0.873	0.952	0.727	0.918	0.787
MLP	Validation	0.917	0.912 (0.852–0.973)	0.964	0.767	0.930	0.868	0.946	0.431

LR, Logistic Regression; ET, Extremely Randomized Trees;MLP, Multi-layer Perceptron; AUC, area under the curve; CI, confidence interval; PPV, positive predictive value; NPV, negative predictive value.

### 3.4 Performance of the clinical model, radiomics model, deep learning radiomics model, and combined model

In the training cohort, the clinical model achieved an accuracy of 0.852, AUC of 0.804 (95% CI 0.7501–0.8570), sensitivity of 0.943, and specificity of 0.591. In the validation cohort, its accuracy was 0.856, AUC was 0.820 (95% CI 0.7372–0.9019), sensitivity was 0.927, and specificity was 0.628.

The radiomics model markedly improved diagnostic performance over the clinical model. In the training cohort, its accuracy was 0.882, AUC was 0.943 (95% CI 0.9167–0.9696), sensitivity was 0.886, and specificity was 0.873. In the validation cohort, its accuracy was 0.917, AUC was 0.912 (95% CI 0.8516–0.9729), sensitivity was 0.964, and specificity was 0.767.

The deep learning radiomics model further improved overall performance. In the training cohort, its accuracy was 0.915, AUC was 0.970 (95% CI 0.9545–0.9858), sensitivity was 0.911, and specificity was 0.927. In the validation cohort, its accuracy was 0.894, AUC was 0.940 (95% CI 0.8956–0.9836), sensitivity was 0.912, and specificity was 0.837.

**Figure 5:**
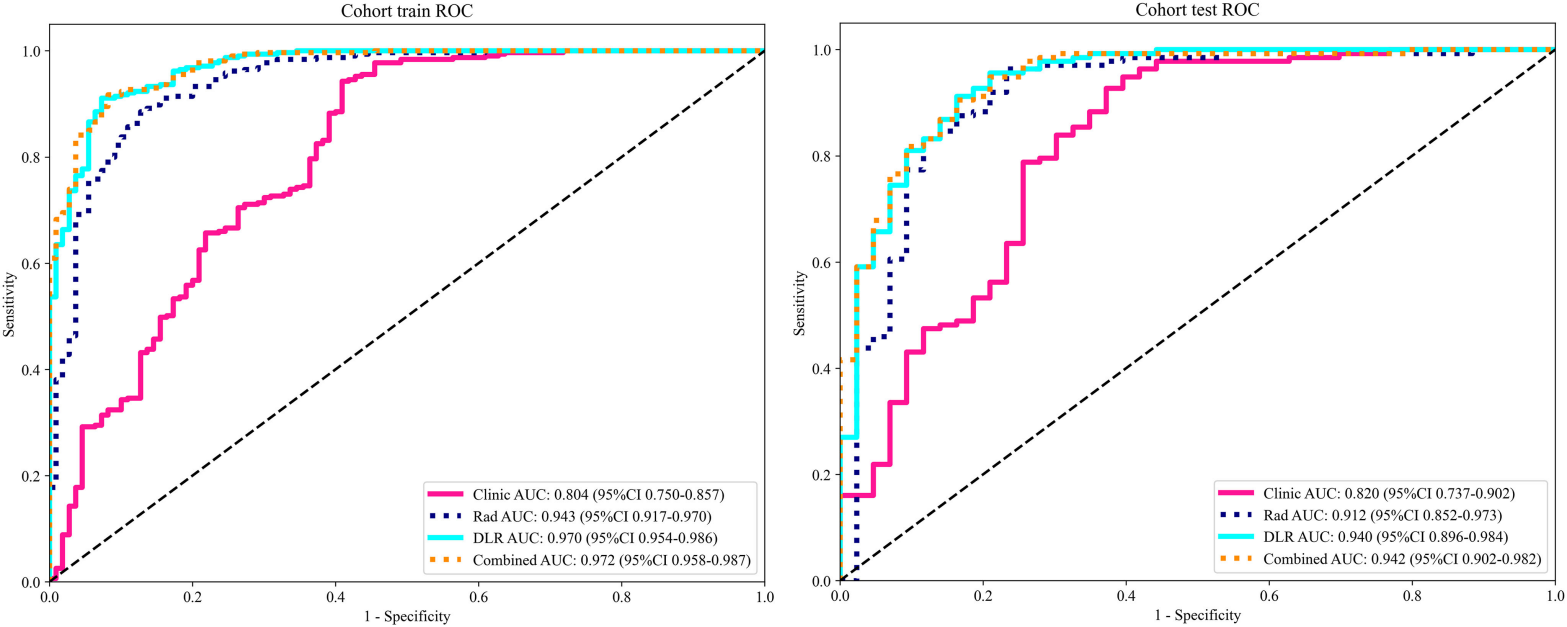
The AUCs of various prediction models in the training and validation cohorts.

**Figure 6:**
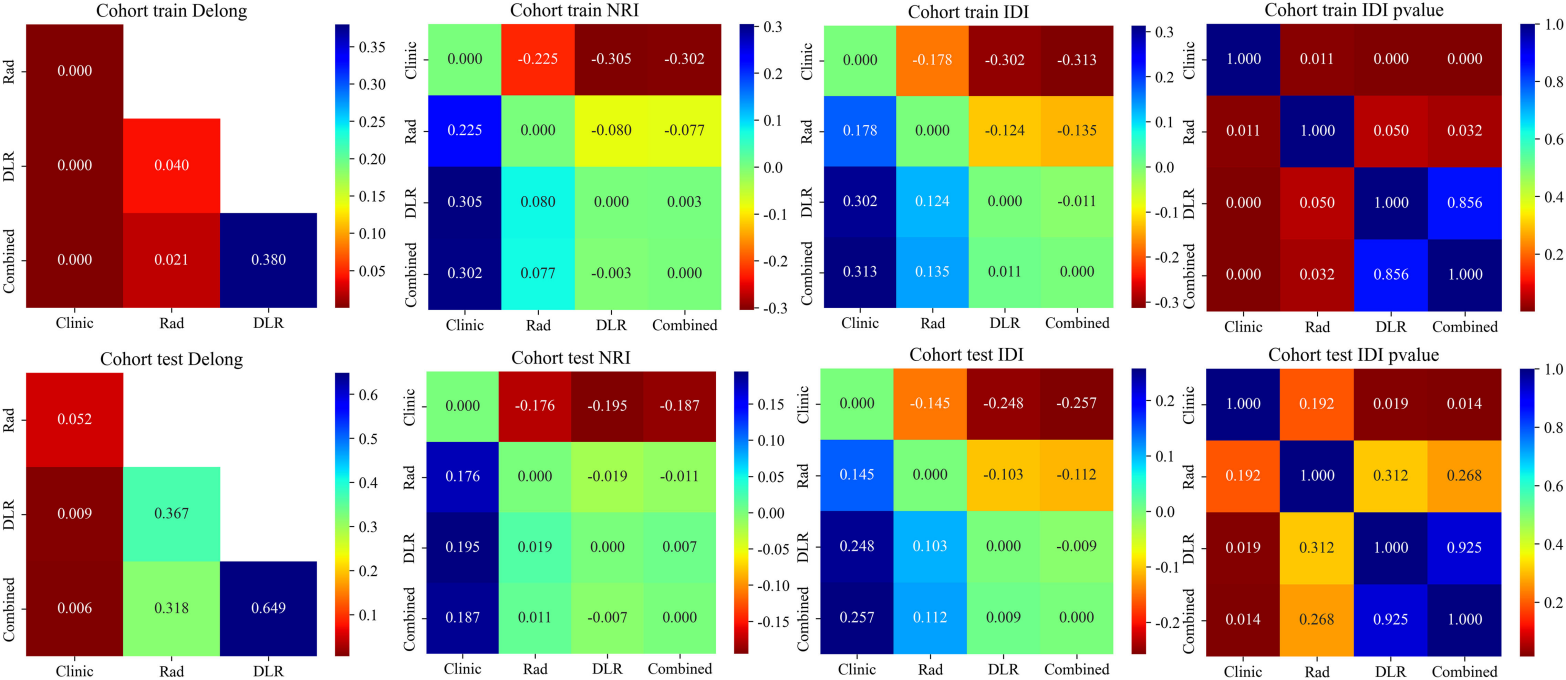
Comparison of the clinical, radiomics, deep learning radiomics, and combined models by DeLong test, NRI, and IDI in the training and validation cohort.

The combined model achieved the highest AUC in both the training and validation cohorts. In the training cohort, its accuracy was 0.918, AUC was 0.972 (95% CI 0.9583–0.9866), sensitivity was 0.917, specificity was 0.918, positive predictive value was 0.970, negative predictive value was 0.795, and F1 score was 0.943. In the validation cohort, its accuracy was 0.889, AUC was 0.942 (95% CI 0.9020–0.9816), sensitivity was 0.905, specificity was 0.837, positive predictive value was 0.947, negative predictive value was 0.735, and F1 score was 0.925 (**Table 4**). The ROC curves of each model are shown in **Figure 5**. In the training cohort, comparisons of predictive performance among the radiomics (Rad), deep learning radiomics (DLR), and combined clinical–radiomics–deep learning (Combined) models using the DeLong test, NRI, and IDI showed that the AUC of the Combined model was significantly higher than that of the Clinic model (DeLong *P* = 0.000) and the Rad model (*P* = 0.021), with IDI (0.313, *P* < 0.001) and NRI (0.302) both indicating significant improvement over the Clinic model; the DLR model also showed a significantly higher AUC than the Clinic model (*P* = 0.000), with IDI (0.302, *P* < 0.001) and NRI (0.305) likewise indicating significant improvement. In contrast, no statistically significant difference in AUC was observed between the DLR and Combined models (*P* = 0.380), and the IDI (0.011, *P* = 0.856) and NRI (0.003) did not suggest a difference in performance. In the validation cohort, the trends were consistent: the AUCs of both the Combined and DLR models were significantly higher than that of the Clinic model (*P* = 0.006 and *P* = 0.009), with IDI values of 0.257 (*P* = 0.014) and 0.248 (*P* = 0.019), and NRI values of 0.187 and 0.195, respectively; no significant differences were found between the DLR and Combined models in AUC (*P* = 0.649), IDI (*P* = 0.925), or NRI, and neither model showed a significant AUC difference compared with the Rad model (*P* = 0.367 and *P* = 0.318), indicating no additional significant predictive gain. Overall, compared with the clinical or radiomics models alone, both the deep learning model and the combined model demonstrated superior predictive performance, with the combined and deep learning models showing comparable performance without significant incremental benefit (**Figure 6**).

**Table 4:** Performance of clinical, radiomics, deep learning radiomics, and combined models.

Model	Cohort	ACC	AUC (95% CI)	SEN	SPE	PPV	NPV	F1-score	Threshold
Clinical	Training	0.852	0.804 (0.750–0.857)	0.943	0.591	0.868	0.783	0.904	0.656
Radiomics	Training	0.882	0.943 (0.917–0.970)	0.886	0.873	0.952	0.727	0.918	0.787
DLR	Training	0.915	0.970 (0.955–0.986)	0.911	0.927	0.973	0.785	0.941	0.842
Combined	Training	0.918	0.972 (0.958–0.987)	0.917	0.918	0.970	0.795	0.943	0.877
Clinical	Validation	0.856	0.820 (0.737–0.902)	0.927	0.628	0.888	0.730	0.907	0.738
Radiomics	Validation	0.917	0.912 (0.852–0.973)	0.964	0.767	0.930	0.868	0.946	0.431
DLR	Validation	0.894	0.940 (0.896–0.984)	0.912	0.837	0.947	0.750	0.929	0.566
Combined	Validation	0.889	0.942 (0.902–0.982)	0.905	0.837	0.947	0.735	0.925	0.537

DLR, Deep learning radiomics; AUC, area under the curve; CI, confidence interval; PPV, positive predictive value; NPV, negative predictive value;ACC, Accuracy;SEN, Sensitivity; SPE,Specificity.

It should be noted that although the DLR model achieved a numerically higher AUC (0.940) than the conventional radiomics model (0.912) in the validation cohort, the difference was not statistically significant (DeLong test *p* = 0.367 and 0.318 for training and validation cohorts, respectively). Similarly, the Combined model did not show a significant AUC improvement over the DLR model (*p* = 0.649). These results indicate that in this dataset, deep transfer learning features did not provide a statistically significant performance gain over conventional hand-crafted radiomics features.

**Figure 7:**
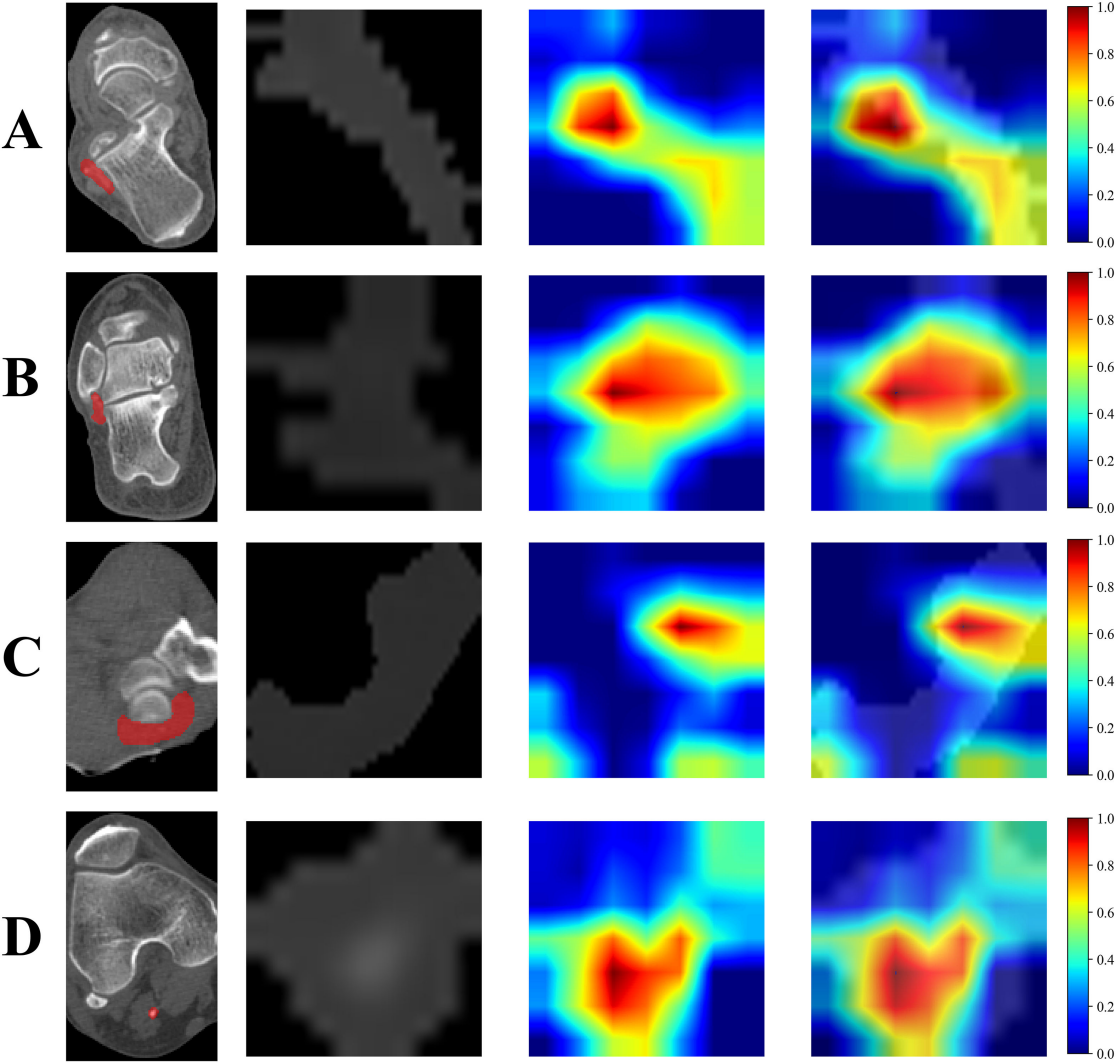
Grad−CAM heatmaps for representative cases. **(A)** True−positive. **(B)** True−negative. **(C)** False−positive. **(D)** False−negative.

To visualize the model’s focus, Grad−CAM heatmaps were generated for representative cases (**Figure 7**). **Figure 7** shows examples of a true−positive, true−negative, false−positive, and false−negative case, respectively.

### 3.5 Construction of a nomogram for the combined model

**Figure 8:**
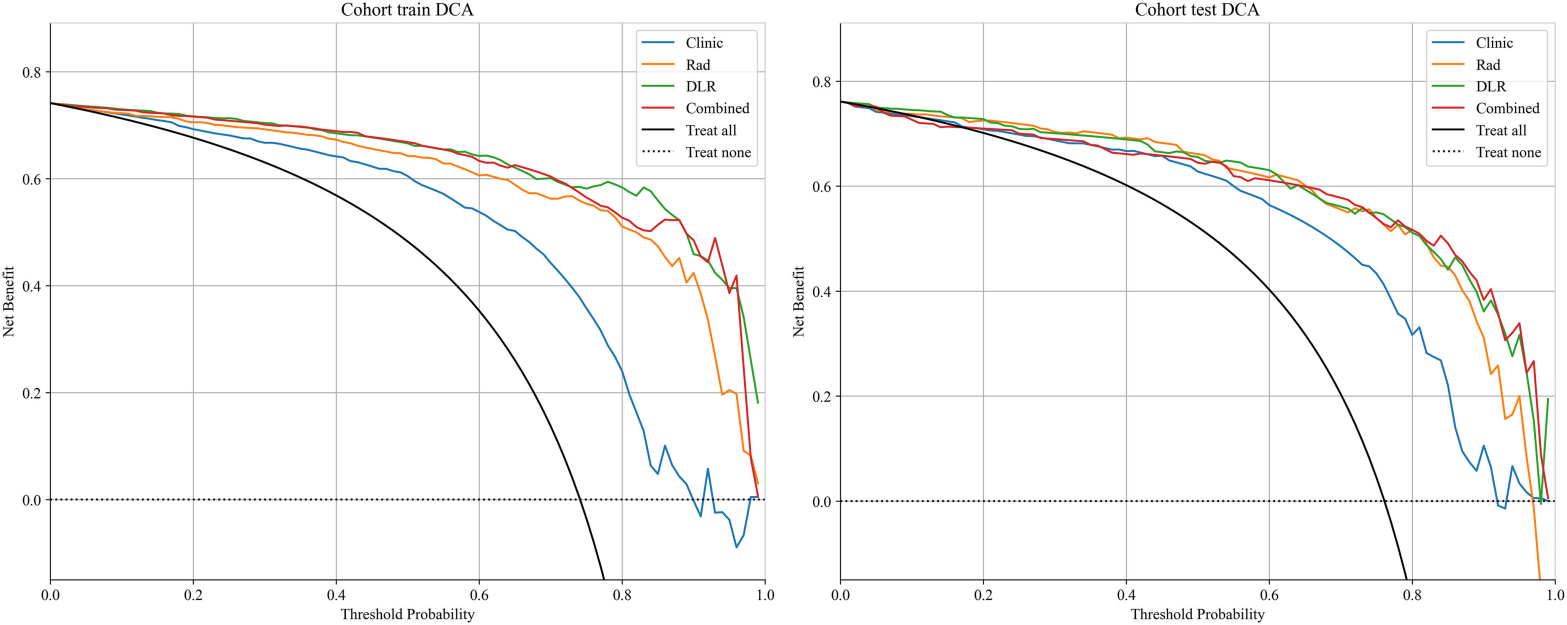
Decision curve analysis was developed with various prediction models.

**Figure 9:**
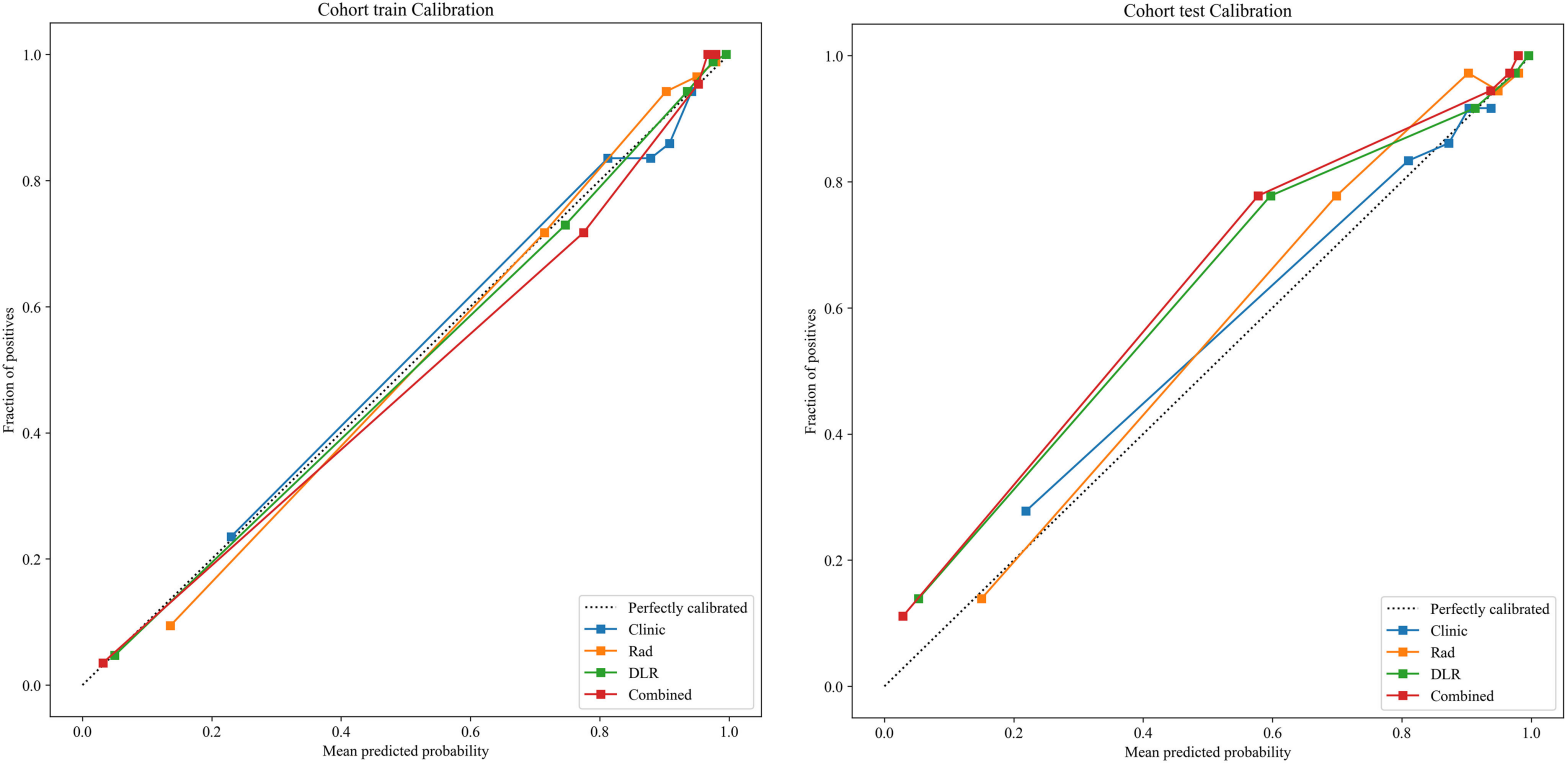
The calibration curve of the radiomics nomogram for the training and validation cohort.

**Figure 10:**
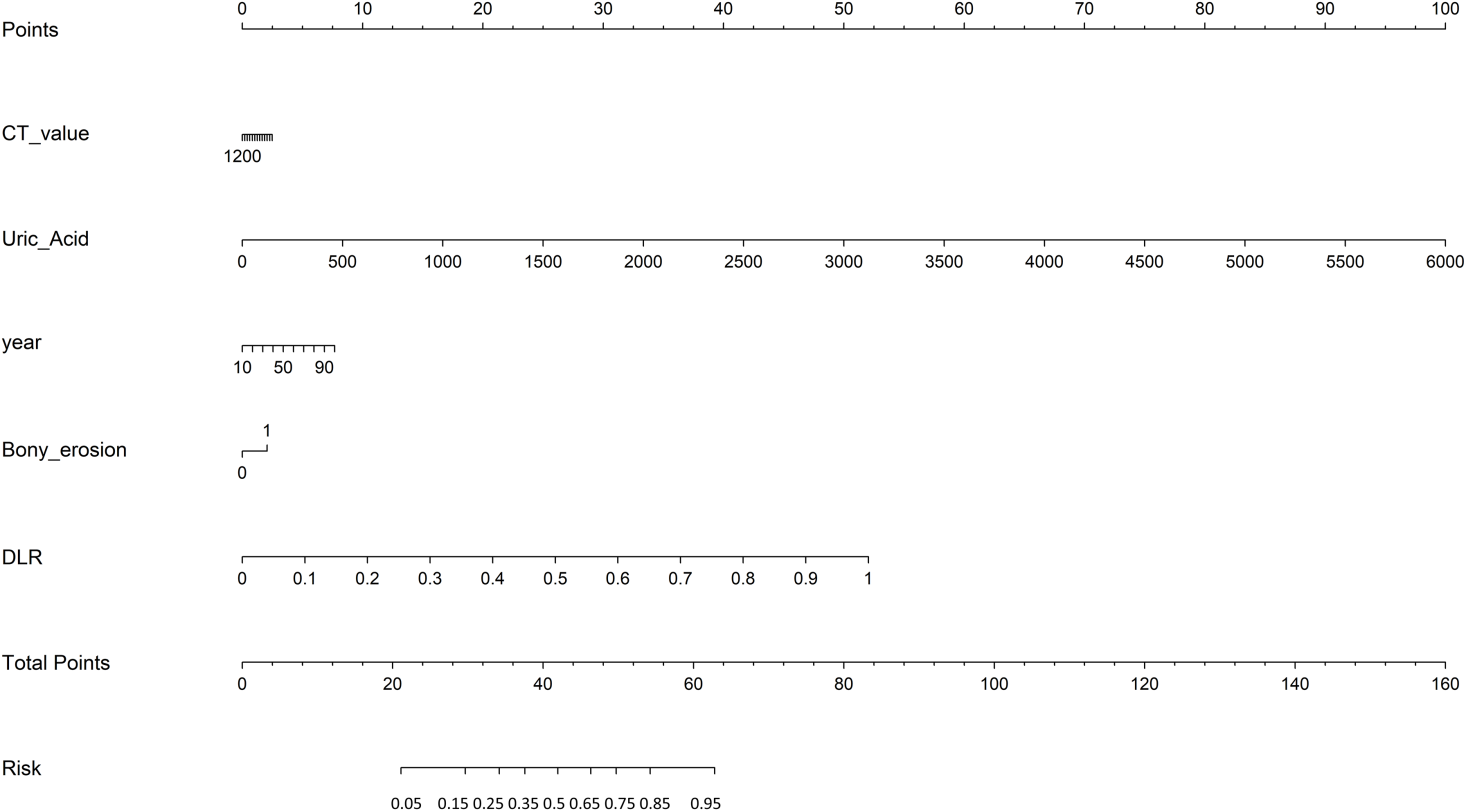
Nomogram incorporating feature fusion and clinical baseline characteristics for the visual identification of MSU.

Comparison of the predictive performance of the four models for MSU crystal deposition showed that the combined model had favorable predictive ability and considerable clinical benefit. Decision curve analysis (**Figure 8**) showed that the deep learning model and the combined model provided higher net benefit than the other three models across a clinically meaningful threshold probability range of 10%-60%.The combined model showed good calibration (**Figure 9**). The Hosmer–Lemeshow test yielded a non−significant *P* value, indicating adequate fit. The nomogram constructed by combining feature fusion with clinical baseline characteristics can be used for the visual identification of MSU (**Figure 10**).

## 4 Discussion

In the 605 patients included in this study, single−energy CT images (135 kVp) CT images contained useful quantitative information for identifying periarticular MSU crystal deposition. The clinical model showed moderate discriminative ability, in which serum uric acid, age, bone erosion, and CT value were independently associated with the outcome. In comparison, the radiomics model showed improved performance over the clinical model. The deep learning radiomics (DLR) model achieved a numerically higher AUC (0.940) than the conventional radiomics model (0.912) in the validation cohort; however, the difference was not statistically significant (DeLong *p* > 0.05). When radiomics features, deep transfer learning features, and clinical variables were further integrated, the combined model achieved the highest validation cohort AUC (0.942), but it did not significantly outperform the DLR model (*p* = 0.649). These findings suggest that, in this dataset, ResNet50-based transfer learning did not provide a statistically significant improvement over conventional hand-crafted radiomics. These findings indicate that multimodal CT modeling may provide a practical non-invasive auxiliary approach for gout evaluation.

We note a reversal in the direction of the association for CT value between univariate (OR 1.001) and multivariate (OR 0.993) analyses. This reversal is likely attributable to confounding by factors such as age, bone erosion, or disease chronicity. In univariate analysis, patients with more advanced disease may have both higher CT value (due to chronic inflammation, fibrosis, or microscopic calcification) and a higher risk of MSU deposition, yielding a positive association. After adjusting for these confounders, the residual independent effect of CT value became slightly negative.

A potential biological explanation for this weak negative association is that MSU crystals themselves have relatively low X−ray attenuation, often lower than surrounding soft tissues or calcified structures (20). In chronic gout, periarticular regions may contain a mixture of crystal deposits, fibrotic tissue, and sometimes calcium salts (21). When the effects of overt bone erosion and age (as a proxy for disease duration) are removed, the remaining signal could reflect the replacement of denser tissue (e.g., fibrotic or calcified tissue) by less dense crystal aggregates (22). However, this is speculative and requires histopathological correlation.

It is also important to acknowledge technical factors that may contribute to the observed association. Partial volume effects, heterogeneity within the ROI, and the inclusion of adjacent non−crystalline soft tissue all affect CT value measurements (23). These factors limit the precision of CT value as a standalone predictor.

Crucially, the magnitude of the effect is extremely small: an OR of 0.993 implies a risk reduction of only 0.7% per one−unit increase in Hounsfield units, which is clinically negligible (24). The statistical significance observed is largely driven by the sample size rather than a meaningful clinical impact (25). Therefore, while CT value reached statistical significance in the multivariate model, it should not be overemphasized as a clinically important predictor. We have removed any statement suggesting that CT value “carries predictive value beyond other covariates.” Future studies with independent cohorts are needed to validate whether this weak association is reproducible or merely a chance finding. The clinical significance of this study lies in addressing unmet needs in the diagnosis of gout. Although the detection of MSU crystals in synovial fluid or tophus aspirate remains the reference standard, aspiration is not always feasible in cases of small-volume periarticular deposits, deep joint involvement, or when patients decline invasive procedures. Imaging therefore plays an important role in clinical decision-making. The 2015 ACR/EULAR gout classification criteria have already incorporated imaging evidence such as the ultrasound double contour sign and DECT-detected urate deposition, reflecting the growing role of imaging biomarkers in gout assessment (3, 5). EULAR recommendations likewise emphasize that the diagnosis of gout should integrate clinical presentation, serum uric acid, synovial fluid analysis when feasible, and imaging findings (26).

The advantage of DECT lies in its ability to directly characterize urate material rather than merely depicting secondary structural changes. Previous studies have demonstrated that DECT has high diagnostic performance for gout; a meta-analysis reported a pooled sensitivity of 84.7%, specificity of 93.7%, and AUC of 0.956 for diagnosing gout (11). Studies using synovial fluid analysis or clinical criteria as the reference standard also support DECT for detecting intra-articular and periarticular urate deposition (9). Nevertheless, DECT is not available in all healthcare facilities, and its sensitivity for early, non-tophaceous lesions may be limited (13). Therefore, if a CT radiomics model is validated externally and proves reliable, it could serve as an auxiliary tool in hospitals without access to DECT or be used to determine whether further DECT examination or invasive crystal confirmation is warranted.

Ultrasound is another important non-invasive imaging tool for gout, capable of showing the double contour sign, tophi, urate aggregates, and bone erosion, and it is low-cost, repeatable, and radiation-free (6), although its diagnostic accuracy depends heavily on the clinician’s experience. Ultrasound can also reflect the dynamic reduction of urate deposits after sustained achievement of normouricemia (27). MRI can assess soft-tissue involvement, synovitis, bone marrow edema, and mass-like tophi, but findings lack specificity and may mimic the imaging appearance of infection or neoplasm (8, 28). By contrast, conventional CT is relatively more accessible and offers good spatial resolution for calcifications, bone erosion, and periarticular deposits. The present results suggest that conventional CT may contain latent quantitative information associated with MSU crystal deposition that can be extracted by radiomics and deep learning methods.

The results of the multivariate analysis have some biological plausibility. Serum uric acid was independently associated with urate deposition, consistent with the core mechanism that urate supersaturation leads to MSU crystal formation. Age was associated with the outcome, possibly reflecting the duration of metabolic exposure and disease chronicity. Bone erosion showed a strong association, consistent with bone and joint destruction caused by chronic crystal deposition and tophaceous inflammation (29–31). The direction of the association for CT value in the multivariate model requires cautious interpretation. CT value values may be simultaneously influenced by crystal composition, density, local tissue environment, partial volume effects, and ROI selection; hence, it should be regarded as a predictive marker rather than a standalone mechanistic explanation.

The radiomics model achieved an AUC of 0.912 in the validation cohort, suggesting that radiomics features can capture meaningful imaging patterns. The final signature included morphological, first-order intensity, and texture features, indicating that MSU crystal deposition may alter not only the mean density but also local heterogeneity and spatial structure. Texture features derived after filtering and transformation may be more sensitive to subtle internal heterogeneity and boundary characteristics that are not easily quantifiable during routine image interpretation. This finding aligns with the fundamental concept of radiomics, namely that medical images contain quantifiable data beyond visual assessment (15).

The deep learning radiomics model attained a validation cohort AUC of 0.940, outperforming the clinical and radiomics models. This improvement suggests that deep features extracted by transfer learning can capture high-level image representations complementary to hand-crafted features. Recent studies have shown that deep learning can improve the differentiation of gout crystals from artifacts on DECT (32). The choice of a ResNet backbone is also consistent with the original residual network framework and subsequent studies on transfer learning for medical imaging (33, 34). For medical imaging data with moderate sample sizes, transfer learning remains a practical strategy, a direction also supported by recent work on foundation model transfer (35). In this study, ResNet50 was used for feature extraction from standardized ROI images and was subsequently fused with hand-crafted radiomics features, resulting in further improved model performance. This outcome is also in line with the broader concept of quantitative imaging phenotyping (36).

The combined model achieved the highest validation cohort AUC of 0.942. Although the increment in AUC over the deep learning radiomics model was small, the combined model maintained high sensitivity while improving specificity compared with the radiomics model alone. Clinically, such a trade-off is meaningful: higher sensitivity helps reduce missed diagnoses, whereas adequate specificity can decrease unnecessary further examinations or treatment escalation. The combined model could serve as an auxiliary decision-making tool for patients with suspected gout, atypical presentations, or ambiguous CT signs. It must be emphasized that this model is not intended to replace synovial fluid analysis or DECT, but rather to provide a generalizable quantitative aid when these methods are unavailable or infeasible.

The MLP (radiomics) model achieved a sensitivity of 0.964 but a specificity of 0.767 in the validation cohort, corresponding to a false−positive rate of 23.3%. The clinical model had an even higher false−positive rate (37.2%). Such false−positive rates are not negligible. In clinical practice, these models should not be used as standalone diagnostic tools. Instead, they may serve as pre−screening aids: a negative prediction is highly reliable (miss rate ~3.6%), whereas a positive prediction should prompt confirmatory testing (DECT or arthrocentesis). The combined model improved specificity to 0.837 (FPR 16.3%), but still carries a non−negligible false−positive burden. Therefore, we no longer describe any model performance as “acceptable” without qualification. Future work should aim to improve specificity, potentially by incorporating additional clinical or imaging biomarkers.

Radiomics features are often criticized as purely statistical variables without biological meaning. To address this, we elaborate the theoretical link between MSU crystal physical properties and CT texture features. MSU crystals have an effective atomic number of approximately 7.7, which is significantly lower than that of calcified tissues (e.g., hydroxyapatite ~9.4) (20). On standard single−energyCT, MSU deposits typically present densities of 160–170 Hounsfield units (HU), whereas calcified lesions exceed 450 HU (37). Consequently, within periarticular MSU deposition regions, voxel intensities are distributed in a pattern determined by the mixture of low−density crystals, fibrotic tissue, inflammatory cells, and occasional punctate calcifications. First−order statistics (e.g., mean, skewness, kurtosis) quantify the overall intensity distribution; the co−existence of crystal aggregates and denser fibrous tissue often yields a positively skewed or leptokurtic distribution. Gray−level co−occurrence matrix (GLCM) features (e.g., contrast, entropy, correlation) reflect spatial relationships between neighboring voxels. Greater crystal heterogeneity or a sharper crystal–soft−tissue interface increases textural contrast and entropy. This chain of reasoning—from crystal physical properties to CT attenuation patterns to texture parameter quantification—provides a theoretical foundation for the biological interpretability of radiomics features in detecting MSU deposition.

In addition to the crystals themselves, the surrounding tissue microenvironment contributes substantially to texture features. Histopathological studies have shown that chronic gouty tophi consist of a central core of MSU crystal aggregates surrounded by a fibrotic capsule, with variable degrees of inflammatory cell infiltration, synovial hyperplasia, and punctate calcification (13, 38). These components—ranging from low−attenuation crystals to higher−attenuation fibrotic and calcified areas—create heterogeneous attenuation patterns that are captured by texture parameters such as GLCM contrast and entropy. While the correspondence between individual texture features and specific histopathological components is not always one−to−one, the aggregate statistical patterns enable machine learning models to distinguish MSU deposition from bone erosion or simple calcification. We acknowledge that radiomics features are data−driven and remain partly “black−box.” Therefore, our model is positioned as a non−invasive screening aid rather than a replacement for histopathological confirmation. Positive predictions should be verified by DECT or arthrocentesis before clinical decision−making. Recent reviews have highlighted that enhancing model interpretability through explainable AI (XAI) methods is a critical direction for radiomics research (39, 40).

This study has several limitations. First, it was a single−center retrospective study. Although we performed internal validation using a held−out validation cohort, external validation across different hospitals, scanner types, and populations is essential to assess the true generalizability of the models. We acknowledge this as a major limitation and plan to conduct multi−center external validation in future work. Second, the reference standard was DECT−identified urate deposition rather than direct MSU crystal detection by polarized light microscopy. Although DECT is widely accepted as a non−invasive imaging tool for gout, it is not the diagnostic gold standard. Notably, DECT has limited sensitivity for early, non−tophaceous, or small−volume urate deposition (21). Consequently, the models developed in this study are inherently designed to detect DECT−positive MSU deposition, not to directly replace the gold standard. In clinical practice, these models should be used as screening tools to identify patients who may benefit from further DECT examination or joint aspiration, but they cannot substitute for definitive gold−standard confirmation. Future studies should incorporate synovial fluid or tophus aspirate confirmation whenever possible to validate the true diagnostic performance of the model. Third, ROI segmentation was performed manually, which may introduce inter−observer variability; automated or semi−automated segmentation could improve reproducibility. Additionally, spectrum bias may exist because we excluded small or ill−defined lesions that prevented reliable ROI delineation. These excluded cases represent early or subtle MSU deposition, where a diagnostic aid would be most valuable. Due to the retrospective design, we could not compare included and excluded cases. Therefore, our model is currently applicable only to well−defined lesions, and its performance on small or ill−defined deposits remains untested. Future studies should include such challenging cases to assess generalizability. Fourth, although the validation cohort was independent of the training process, prospective studies are still needed to evaluate the model’s performance and impact on decision−making in real clinical workflows. Fifth, calibration curves and decision curve analyses require further validation in external or prospective cohorts to establish clinically applicable thresholds. Sixth, hand−crafted features used whole−lesion ROIs, whereas deep features used only the maximum cross−sectional ROI because ResNet50 requires fixed 2D input. This inconsistency sacrifices 3D spatial information (e.g., crystal volume, longitudinal extent), a limitation we acknowledge. Future studies should consider 3D−CNNs. Seventh, although we have established a theoretical link between CT texture features and MSU deposition pathology, direct histopathological validation of the specific selected radiomics features was not performed. This limits the biological interpretability of our model and should be addressed in future studies using co−registered imaging−pathology datasets. Additionally, subgroup analyses across different anatomical sites, deposit sizes, tophaceous status, and non−MSU diagnoses were not performed due to limited sample size, and the model’s robustness in these subgroups remains untested. Eighth, although the images used in this study were 135 kV single−energy images comparable to those acquired on standard single−energyCT scanners, all acquisitions were performed on a DECT scanner operating in dual−energy mode. We did not perform external validation on truly independent conventional single−energy CT scanners. Therefore, the generalizability of our models to non−DECT scanners remains to be verified. Future studies should include a separate cohort scanned on a standard single−energy CT scanner to confirm the robustness of the models.

We acknowledge that the multi−step feature selection pipeline (t−test, Pearson correlation, LASSO, mRMR) may be considered over−engineering and could potentially lead to unstable feature selection, especially with a moderate sample size. To mitigate this risk, we restricted all selection steps to the training cohort and used cross−validation for LASSO parameter tuning. Nevertheless, the stability of the selected features was not formally assessed (e.g., by bootstrap or repeated cross−validation), which is a limitation of this study. Future work should consider simpler or more stable selection methods, such as embedded regularization alone or stability selection with bootstrapping.

Despite these limitations, this study demonstrates that quantitative conventional CT imaging can be used for the non-invasive detection of periarticular MSU crystal deposition. Because conventional CT is more readily available than DECT in many healthcare settings, if the combined model proves robust after external validation, it could assist radiologists and clinicians in patient stratification, selecting candidates for DECT or aspiration, and informing early intervention.

## 5 Conclusions

In this single-center retrospective study of 605 patients, both radiomics and deep learning radiomics models based on single−energy CT (135 kVp) improved the detection of periarticular MSU crystal deposition compared with a clinical model alone. However, the deep learning radiomics model did not show a statistically significant advantage over the conventional radiomics model. The combined model achieved the highest AUC but did not significantly outperform the deep learning radiomics model. The combined model integrating clinical variables, hand-crafted radiomics, and deep learning features achieved the highest AUC in the validation cohort (0.942) and showed relatively balanced sensitivity and specificity. Nevertheless, the moderate specificity (0.767–0.837) results in a false−positive rate of 16.3%–23.3%, which is not negligible. Therefore, these models should be used as screening aids rather than confirmatory diagnostic tools; positive predictions require further confirmation. External validation, prospective studies, and clinical workflow assessment are warranted.

## Data Availability

The original contributions presented in the study are included in the article/supplementary material. Further inquiries can be directed to the corresponding authors.
